# Tomato Processing By-Products as a Sustainable Source of Lycopene and Other Bioactive Compounds for Animal Nutrition: A Circular-Economy Perspective

**DOI:** 10.3390/molecules31152589

**Published:** 2026-07-24

**Authors:** Vasfiye Kader Esen, Dilek Öğdüm, Selim Esen

**Affiliations:** 1 Department of Breeding and Genetics, Sheep Breeding Research Institute, General Directorate of Agricultural Research and Policies, Balıkesir 10200, Türkiye; vasfiye.esen@tarimorman.gov.tr; 2 Sheep Breeding Research Institute, General Directorate of Agricultural Research and Policies, Balıkesir 10200, Türkiye; dilek.ogdum@tarimorman.gov.tr; 3 Balıkesir Directorate of Agriculture and Forestry, Republic of Türkiye Ministry of Agriculture and Forestry, Balıkesir 10470, Türkiye

**Keywords:** tomato pomace, lycopene, carotenoid, bioactive compound, circular economy, animal nutrition, antioxidant, Nrf2, ensiling, agro-industrial by-product

## Abstract

Tomato (*Solanum lycopersicum* L.) is the world’s second-most cultivated vegetable. Production reached 192 million tonnes in 2023; approximately 23% is industrially processed, leaving 4.3 to 10.2 million tonnes of pomace each year. Despite its high content of lycopene, tocopherols, polyphenols, dietary fibre, and seed oil, most of this residue is composted, landfilled, or fed without prior processing. This narrative review examines how the chemistry of tomato by-products maps onto their effects in farm animals, and how green-extraction biorefinery fits within the European Green Deal. Reported pomace lycopene runs from 36.7 to 50.2 mg/100 g DM, with phenolics near 161.8 mg GAE/g. Lycopene is an efficient singlet-oxygen quencher; it activates Keap1–Nrf2–ARE signaling while dampening NF-κB. Supercritical CO_2_, ultrasound, microwave, pressurized-liquid, and NADES extractions can now recover up to 91% of peel lycopene using green, food-grade solvents. In broilers, 5 to 10% pomace or 30 to 400 mg/kg purified lycopene improves antioxidant status and lessens heat stress; in dairy ruminants, ensiled pomace at 10 to 40% maintains milk yield while improving milk PUFA. In finishing pigs and rabbits, dietary pomace or lycopene improves tissue oxidative stability, with growth benefits reported in heat-stressed rabbits. Standardized bioactive reporting and clearer inclusion limits remain the main research priorities.

## 1. Introduction

The global livestock sector is being reshaped by three converging pressures. First, demand for animal-source food is projected to rise by nearly 70% by 2050 [1]. Second, climate change is amplifying heat stress in production environments. Third, regulatory commitments to circular resource use are being tightened under the European Green Deal and the United Nations Sustainable Development Goal 12.3 on food loss and waste [1,2,3]. Against this background, agro-industrial by-products have become increasingly central to sustainable feed formulation. The European Feed Manufacturers’ Federation (FEFAC) has reported that practically none of the raw materials used in EU compound-feed production are of food-grade quality. Taken together, these observations indicate that circular feeding is no longer a marginal practice but has become the dominant operational paradigm of the sector [4]. The projected increase in demand warrants some caution, however, since the expanding market for plant-based and cultivated-meat products, together with the growth of vegetarian dietary patterns, may moderate the rate of increase even where aggregate demand continues to rise.

Tomato (*Solanum lycopersicum* L.) is the second-most cultivated vegetable worldwide, and a substantial share of the crop is industrially processed into paste, juice, and related products [5,6,7]. Processing yields a solid residue, commonly termed tomato pomace, which comprises peel, seeds, and residual pulp in proportions varying by cultivar, processing technology, and ripeness, and which represents 3–5% by weight of the raw fruit [8].

Historically, tomato pomace has been treated as a low-value disposal stream, frequently sent to landfills or, where better managed, used as a non-formulated feed or compost substrate. This reflects a paradigm of waste rather than co-product. Yet the residual matrix retains a high concentration of bioactive molecules: lycopene concentrations of 36.7–50.2 mg/100 g dry matter [9], total polyphenol content averaging 161.8 mg GAE/g, total flavonoid content of 30.6–378.7 mg quercetin equivalents/g, and DPPH radical-scavenging activities of 29.9–75.0% [9]. The seed fraction contributes high-quality oil (mainly linoleic and oleic acids) and a protein concentrate rich in essential amino acids [10,11]. The peel fraction concentrates approximately five-fold more lycopene than the pulp [12] and harbours a cutin-rich cuticle (40–85% *w*/*w* cutin [8]) that now attracts attention as a bio-polyester precursor [13,14].

Lycopene, the dominant carotenoid responsible for the characteristic red pigmentation, is of particular interest because of its exceptional singlet-oxygen-quenching capacity (more than two-fold greater than β-carotene and ~100-fold greater than α-tocopherol on a molar basis) [15] and its capacity to modulate transcription factors regulating the cellular antioxidant response [16,17]. A substantial body of work in food and natural-product chemistry has examined the isomerization, encapsulation, and structure–activity relationships of lycopene [18,19], while a parallel body of evidence in animal science has accumulated regarding the effects of dietary lycopene supplementation on production performance, oxidative status, and product quality across livestock species [9,20,21]. These two bodies of work have largely developed in parallel. The food-chemistry literature has concentrated on the isomerization and extraction of lycopene [18,19], whereas the animal-science literature has documented production responses to dietary supplementation [9,20,21], and existing by-product reviews have generally considered tomato pomace alongside other agro-industrial residues rather than in relation to its lycopene chemistry. A synthesis that follows the compound from the residual matrix, through recovery and preservation, to its effects in the animal has not previously been assembled, and it is this gap that the present review seeks to address.

**Figure 1:**
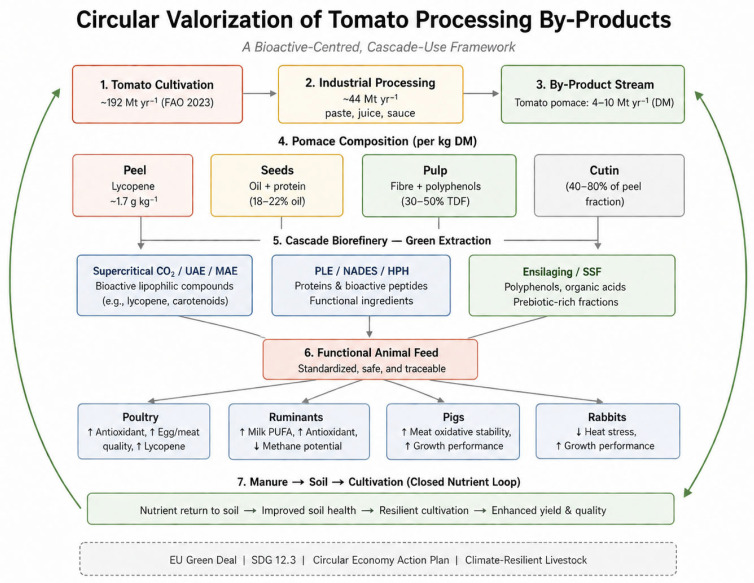
Conceptual framework of the circular valorization of tomato processing by-products. The diagram integrates the cascade-use principle from agro-industrial input (cultivation → processing → by-product stream) through compositional partitioning (peel-derived lycopene; seed-derived oil and protein; pulp-derived fibre and polyphenols), green-extraction biorefinery (SFE-CO_2_, UAE, MAE, PLE, NADES, HPH, ensiling, solid-state fermentation), application as functional animal feed across species (poultry, ruminants, pigs, rabbits), and closure of the nutrient cycle through manure return to soil. The framework explicitly references the policy context of the European Green Deal, SDG 12.3 on food loss and waste, the proposed EU Circular Economy Act, and climate-resilient livestock production ↑: increase; ↓: decrease.

Even with this growing body of work, three gaps motivate the present narrative review. First, the chemistry of lycopene, including all-E/Z isomerization, oxidative degradation, and matrix-dependent bioaccessibility, is rarely integrated with the in vivo outcomes observed in livestock, with reviews instead bifurcating along disciplinary lines [18,22]. Second, ensiling and other lactic-acid-bacterial preservation strategies for tomato pomace have not been systematically connected to the retention of bioactive integrity during storage and rumen passage [23,24]. Third, the circular-economy framing of tomato by-product valorization remains largely conceptual; the technical inclusion limits, dose–response patterns, and species-specific responses are scattered across small primary studies. The overall aim of this review is therefore to bring together the chemistry of tomato by-products and their documented effects in livestock, which have to date been treated largely in isolation. The present review therefore aims to (1) characterize the chemical and bioactive composition of tomato pomace; (2) summarize molecular mechanisms by which lycopene modulates redox and inflammatory pathways relevant to animal physiology; (3) compare conventional and green recovery technologies; (4) synthesize the published in vivo evidence for tomato pomace and lycopene in poultry, ruminants, pigs, and rabbits; and (5) situate these findings within the European Green Deal circular bioeconomy framework, with explicit attention to Mediterranean production contexts. A visual overview of this conceptual framework, illustrating the cascade from agro-industrial by-products to functional animal feed and the subsequent closure of the nutrient cycle, is presented in Figure 1.

## 2. Tomato Industry: Global Production and By-Product Generation

Tomato production reached 192 million tonnes globally in 2023 with a compound annual growth rate of 1.5% [5]. China leads with over 70 million tonnes (an estimated 11% processed), followed by India (1% processed), Türkiye (20% processed), the United States, and Italy [5,6]. Of the 44.2 million tonnes processed industrially in 2023, the dominant outputs are paste, sauce, juice, and canned products [6]. The Mediterranean basin, which includes Italy, Spain, Greece, Türkiye, Tunisia, and southern France, accounts for a disproportionate share of processing capacity and therefore concentrates by-product generation within a relatively confined geographic area suited to circular regional integration [6,7].

Tomato processing waste is typically partitioned into three streams: peel (skin), seeds, and residual pulp, with the relative proportions varying between cultivars and processing technologies. Pomace generally represents 4–10% of fresh fruit weight under conventional hot-break processing, but can reach 10–30% w/w in finishing operations such as juice or paste manufacture [8,13,25]. Globally, an estimated 4.3–10.2 million tonnes of tomato processing residue are generated annually [6,7,8,25]. Inadequate management of this stream creates three problems: (i) direct landfill cost and methane emissions from anaerobic decomposition of fermentable material; (ii) leachate generation with high biochemical oxygen demand owing to residual sugars and organic acids; and (iii) opportunity cost from non-recovery of high-value compounds [8,9,26]. At the systems level, biophysical modelling indicates that EU-wide adoption of circular feeding could reduce agricultural land use by up to 42% and per-capita agricultural greenhouse-gas emissions by up to 31% while still meeting protein demand [27,28,29].

## 3. Chemistry and Composition of Tomato Pomace

Among the major agro-industrial residues, including apple, citrus, olive, grape, and brewer’s spent grain, tomato pomace is particularly notable for the high concentration of intact bioactive compounds that are retained through processing. Its composition is, however, highly heterogeneous, since cultivar, processing temperature, and the finishing and deseeding steps each influence the chemical profile of the final residue. Representative compositional ranges have been compiled by Lu et al. [9]. According to published surveys, dried pomace contains 150–230 g/kg crude protein, 50–200 g/kg crude fat, and 250–510 g/kg total dietary fibre (Table 1). The seed fraction provides the majority of the protein and the linoleic acid-rich lipid pool [10,11,30], whereas the peel fraction provides the majority of the dietary fibre and concentrates carotenoids approximately five-fold relative to the pulp [12,14]. With respect to the mineral profile reported by Becze et al. [31], potassium and phosphorus are the dominant elements, following the K  >  P  >  Ca  >  Mg order typically observed in plant-derived residues. The lipid fraction is dominated by polyunsaturated fatty acids, which account for 55–62% of total fatty acids, with linoleic acid as the principal species. Of these sources of variation, the processing route appears to exert a stronger influence on the final bioactive profile than cultivar alone [32]. The choice between cold-break and hot-break processing, and the presence or absence of finishing and deseeding steps, largely determines the retained carotenoid and phenolic content, yet these conditions are among the least consistently reported, which limits the reproducibility of published compositional data. Consistent with this, a recent comparison of three San Marzano genotypes found that, although the proximate composition was largely conserved, the flavonoid profile and total antioxidant capacity differed appreciably between genotypes, confirming that the phenolic fingerprint is determined genetically as well as by processing [33]. In comparison with other agro-industrial residues, tomato pomace is distinguished less by its protein or fibre content, which is broadly comparable to that of grape and citrus pomace, than by its lycopene concentration, for which there are few parallels among common by-products. A further limitation of the available literature is the absence of a shared analytical convention; concentrations reported on an extract, fresh-weight, or dry-matter basis are not directly comparable, and this heterogeneity constrains any quantitative synthesis.

The bioactive content is the principal feature distinguishing this matrix. Lycopene concentrations reported across the modern literature [9] place dried pomace among the most concentrated known natural sources of the carotenoid, and β-carotene, lutein, phytoene, phytofluene, and the tocopherols complete the lipid-soluble pool. The polyphenol fraction is comparable in magnitude to that of many specialty fruit extracts [9] and comprises constituents of widely differing polarity, among them naringin and naringenin from the flavanone family, quercetin and rutin from the flavonols, and caffeic, chlorogenic, and p-coumaric acids from the hydroxycinnamic group [31,34] giving the matrix the characteristic phenolic fingerprint. The flavanone and flavonol aglycones, although frequently grouped with the water-soluble constituents, are in fact only sparingly soluble in water, whereas the hydroxycinnamic acids are appreciably more polar. Solubility therefore varies markedly across this group, and this variation governs the behaviour of each class during extraction and digestion.

## 4. Chemistry and Molecular Mechanisms of Lycopene Action

### 4.1. Lycopene Chemistry

The bioactivity of lycopene is fundamentally determined by its molecular structure. The acyclic tetraterpene C40H56 contains thirteen carbon–carbon double bonds, of which eleven are conjugated, and this extended π-electron system accounts for both its characteristic red colour (λmax ≈ 472 nm) and its exceptionally high capacity to quench singlet oxygen [35,36]. The conjugated backbone also permits geometric isomerization, and the position-dependent thermodynamics of this isomerization largely govern the behaviour of the molecule from harvest to consumption.

In the fresh fruit, roughly 90% of lycopene exists as the all-E (trans) isomer, a planar and highly crystalline form whose bioavailability is poor [37]. In animal plasma after consumption, more than half of total lycopene appears as Z (cis) isomers, and the literature on absorption [18,38,39] broadly converges on the underlying reason: bent Z-geometries solubilize more readily in bile micelles, are less prone to recrystallization, and are incorporated into chylomicrons more readily than the planar all-E form. The site of isomerization is similarly relevant. Industrial heat processing of paste and sauce drives a measurable trans-to-cis shift [18,40]. A similar shift is induced by the acidic gastric environment [38], and naturally occurring polysulfides and isothiocyanates in some companion foods further catalyse the conversion [40]. These observations have direct implications for feed formulation: the matrix and the choice of pretreatment are of comparable importance to the absolute lycopene content itself. Comminution to break cellular structures, mild heating to drive a fraction of the lycopene into Z-forms, and the presence of dietary lipid as a micelle vehicle all measurably improve the amount of lycopene reaching tissues [36,37,41]. Incorporating pomace as a bulk ingredient, without such pretreatment, leaves much of its lycopene bioinaccessible.

### 4.2. Direct Antioxidant Activity

Lycopene is the most potent singlet-oxygen quencher among dietary carotenoids, with a quenching rate constant approximately two-fold greater than β-carotene and roughly 100-fold greater than α-tocopherol on a molar basis [15,21]. The mechanism involves physical quenching, in which singlet-oxygen energy is dissipated as heat through the relaxation of the carotenoid triplet excited state, and chemical quenching, in which the carotenoid is consumed by oxidative cleavage [21,42]. The extended conjugated polyene system also confers efficient scavenging of peroxyl radicals (ROO−), peroxynitrite (ONOO−), and hydroxyl radicals (−OH), placing lycopene among the principal lipid-phase antioxidants in animal tissues [21,43]. The activity of lycopene is not restricted to radical chemistry. It has also been reported to enhance gap-junction intercellular communication, to modulate apoptotic and cell-cycle signaling, and to influence lipid-regulatory transcription factors, which indicates that its biological role extends beyond the maintenance of redox balance [20,21].

### 4.3. Keap1–Nrf2–ARE Signaling

A second mechanism, often more important than direct radical scavenging, is the activation of the Kelch-like ECH-associated protein 1 (Keap1)/nuclear factor erythroid 2-related factor 2 (Nrf2)/antioxidant response element (ARE) pathway [16,17,44]. Under basal conditions, Nrf2 is retained in the cytoplasm by its negative regulator Keap1 and targeted for Cullin 3-dependent ubiquitination and proteasomal degradation [45,46]. Oxidative or electrophilic stress, or interaction with bioactive phytochemicals including lycopene, modifies critical cysteine residues on Keap1 that have been implicated in electrophile sensing (notably Cys151, Cys273, and Cys288 in the most-studied contexts, with additional residues such as Cys226 and Cys297 reported under different electrophile classes), disrupting Keap1-mediated Nrf2 ubiquitination [45]. The accumulated free Nrf2 translocates to the nucleus, dimerizes with small Maf proteins, and binds to the ARE in the promoter regions of cytoprotective genes including superoxide dismutase 2 (SOD2), glutathione peroxidase (GPx), NAD(P)H quinone dehydrogenase 1 (NQO1), heme oxygenase 1 (HO-1), and γ-glutamylcysteine ligase (GCL) [45,47]. A complementary mechanism involves the p62 (sequestosome-1) protein, which competes with Nrf2 for the Keap1-binding site and channels Keap1 to autophagic degradation, thereby establishing a positive-feedback amplification of the antioxidant response [45,48]. The Keap1–Nrf2–ARE induction described above is attributable principally to lycopene. Several polyphenols present in whole pomace [31,34], notably quercetin, naringenin, and the caffeic and chlorogenic acids, have themselves been reported to activate Nrf2 [49], and are therefore likely to contribute to this response when whole pomace, rather than purified lycopene, is supplied.

In broilers, dietary lycopene at 20–30 mg/kg upregulates hepatic mRNA expression of Nrf2, SOD2, NQO1, and HO-1 and increases circulating glutathione peroxidase activity [16]. In intestinal epithelium challenged by the mycotoxin deoxynivalenol, lycopene restored Nrf2 nuclear translocation, repaired barrier function (as measured by transepithelial electrical resistance and tight-junction protein expression), and reduced diamine oxidase release [17]. In aging-induced ovarian oxidative stress in laying hens, lycopene applied to cultured granulosa cells at 100 ng/mL activated the Nrf2/HO-1 axis, increased antioxidant-enzyme activities, and inhibited granulosa cell apoptosis [50].

### 4.4. NF-κB and Inflammatory Crosstalk

The Nrf2 pathway and the nuclear factor κB (NF-κB) inflammatory pathway exhibit reciprocal regulation: Nrf2 activation suppresses NF-κB-driven pro-inflammatory gene expression, while sustained NF-κB activity attenuates Nrf2 nuclear translocation [51]. Lycopene supplementation in heat-stressed broilers and quails downregulates NF-κB and the expression of associated pro-inflammatory cytokines while upregulating Nrf2 and downstream antioxidant enzymes [21,52,53]. Through a series of studies in Japanese quails and broiler chickens, Sahin et al. [21] have established that the dual modulation of NF-κB (suppression) and Nrf2 (induction) is the most consistent transcriptional signature of dietary lycopene under thermal challenge, accompanied by reductions in muscle and serum malondialdehyde (MDA) and increases in muscle lycopene deposition [52,53]. Lycopene is not, however, the only antioxidative constituent of the matrix. The polyphenol fraction contributes direct radical scavenging and metal chelation, whereas the dietary fibre reduces the oxidative load indirectly, both by binding bile acids and through its fermentation to short-chain fatty acids in the hindgut. Whether these constituents act additively, synergistically, or antagonistically with lycopene when whole pomace is fed remains to be established, and clarifying these interactions represents a clear priority for mechanistic study [9,31].

### 4.5. Lipid Metabolism

**Figure 2:**
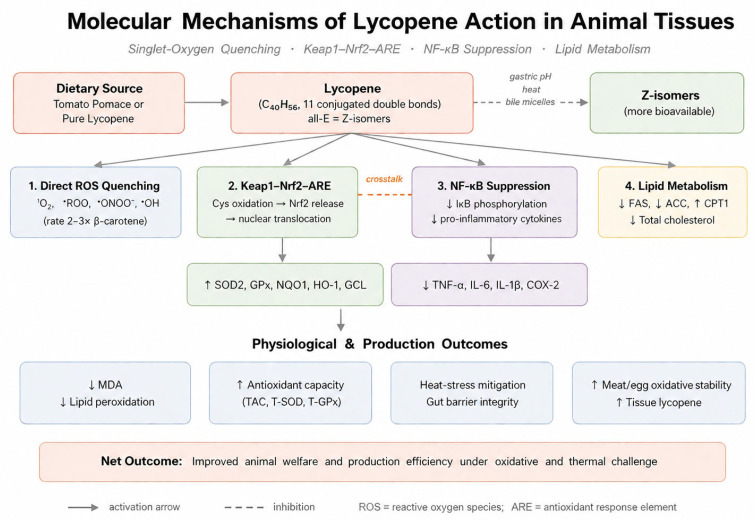
Molecular mechanisms of lycopene action in animal tissues. Four converging arms describe (1) direct quenching of reactive oxygen species (^1^O_2_, peroxyl radicals ROO−, peroxynitrite ONOO−, and hydroxyl −OH), with a quenching rate constant more than 2× that of β-carotene; (2) activation of the Keap1–Nrf2–ARE pathway through modification of critical cysteine residues on Keap1, releasing Nrf2 for nuclear translocation and inducing antioxidant gene expression (SOD2, GPx, NQO1, HO-1, GCL); (3) reciprocal suppression of NF-κB signalling with downregulation of pro-inflammatory cytokines (TNF-α, IL-6, IL-1β, COX-2); and (4) lipid metabolism modulation (↓ FAS, ↓ ACC; ↑ CPT1; reduced total cholesterol). The downstream consequences include reduced MDA, increased total antioxidant capacity, heat-stress mitigation, and enhanced product oxidative stability.

Lycopene also modulates lipid metabolism in monogastric animals. Wan et al. [54] demonstrated in broilers that lycopene supplementation reduces abdominal fat deposition and lowers hepatic expression of lipogenic genes including fatty acid synthase (FAS) and acetyl-CoA carboxylase (ACC) while inducing carnitine palmitoyltransferase 1 (CPT1) and PPARα-mediated β-oxidation. In laying hens, dietary lycopene at 10–20 mg/kg or tomato powder at 5–10 g/kg did not consistently alter the serum lipid profile but increased yolk lycopene and carotenoid deposition and reduced serum and egg-yolk malondialdehyde [55,56]. In finishing pigs, dietary lycopene at 20 mg/kg did not alter circulating cholesterol but lowered MDA concentrations in pork belly, indicating preferential effects on tissue oxidative stability rather than systemic lipid homeostasis [57]. These four mechanisms are not independent. Singlet-oxygen quenching and Nrf2-mediated enzyme induction together lower the tissue oxidative load; suppression of NF-κB restrains the inflammatory response elicited by heat and mycotoxin challenge; and the accompanying shift in lipid metabolism directs substrate away from fat deposition. Taken together, these actions provide a mechanistic basis for the lower malondialdehyde concentrations, the improved antioxidant status, and the greater tolerance of heat stress that are reported in the feeding trials considered below. These mechanisms are summarized in Figure 2; their consequences for animal performance are examined, species by species, in Section 7.

## 5. Recovery Technologies for Bioactive Compounds

The development of lycopene-recovery methods for tomato by-products closely parallels the broader shift toward green-chemistry extraction. The conventional approach relies on organic solvents such as hexane, ethanol, ethyl acetate, dichloromethane, or chloroform–methanol mixtures, applied with heat to disrupt the cuticular barrier. Solvent extraction has been widely applied [8,58,59], but its limitations are well documented: low yields owing to the recalcitrance of the tomato peel cuticle (which can be 40–85% cutin by weight), large solvent volumes with associated environmental and worker-safety costs, thermal degradation, unwanted isomerization, and residues that disqualify the extract from food and nutraceutical applications where tight regulatory limits apply [8,13,60].

The technologies that have displaced solvent extraction in modern biorefinery designs are reviewed in detail by Eslami et al. [8]. Supercritical fluid extraction with carbon dioxide above its critical point (Tc = 31.1 °C, Pc = 7.38 MPa, with practical working conditions typically at 200–450 bar and 40–80 °C) is the most established commercial alternative. The CO_2_ acts as a tunable non-polar solvent, dissolves the carotenoids cleanly, and leaves no residue when depressurized; yields of up to 31.4 mg lycopene per 100 g dry matter, representing ~90% of the total extracted carotenoids, have been reported under optimized conditions (≈460 bar, 80 °C) [61], with positive net present value and competitive payback times at industrial scale [62]. Ultrasound- and microwave-assisted methods rely on a different mechanism. Acoustic cavitation and dielectric heating disrupt the cellular structures that physically retain the lycopene, and the green-solvent work of Drosou et al. [63] using ethyl lactate as the medium achieved 91% extraction yield from peel under modest power inputs. High-pressure homogenization quadruples the lycopene released from aqueous tomato peel suspensions, from around 14% to over 56%, and simultaneously raises protein, polyphenol, and antioxidant capacity recovery by comparable margins [64]. Pulsed electric fields, natural deep eutectic solvents [65], and pressurized liquid extraction with sunflower oil as the carrier, which produces a lycopene-enriched oil directly, with no transfer step needed [63], complete the current methodological repertoire. Green-solvent extraction has continued to advance; a volatile natural deep eutectic solvent (menthol:thymol) applied under ultrasound has recently been shown to recover lycopene from tomato peel waste as a fully food-grade, hexane-free process [66]. These technologies, together with their operating conditions, reported lycopene yields, and principal advantages and limitations, are compared in Table 2.

The most integrated configuration is the cascade biorefinery, in which a single pomace input feeds a sequence of extraction steps that recover lycopene, β-carotene, polyphenols, seed oil, dietary fibre, pectin, cutin, and protein as distinct co-product streams [58,65]. The life-cycle assessment work of Eslami et al. [6] on the Italian tomato sector identifies biorefinery integration as the dominant lever for reducing the carbon footprint of paste and sauce production, a conclusion that aligns with the European Green Deal’s circular framework [67]. Enzyme-aided extraction has been used to loosen the recalcitrant peel cuticle prior to supercritical-CO_2_ recovery, raising the lycopene concentration of the freeze-dried matrix by roughly 150%, with total extracted lycopene about three-fold higher than in the untreated control [68]. Solid-state fermentation with *Lactobacillus* and *Saccharomyces* strains has separately been shown to enzymatically loosen the matrix, reduce crude fibre, and raise the extractability of the carotenoid fraction before the main extraction steps [69]. These technologies differ considerably in their technological maturity. Supercritical-CO_2_ extraction is already applied at commercial scale, ultrasound- and microwave-assisted extraction have reached pilot to early-commercial stage, and pulsed electric fields and systems based on natural deep eutectic solvents remain largely at laboratory scale. The principal barriers to industrial adoption are therefore economic and infrastructural rather than technical, and include the capital cost of the equipment, the difficulty of matching throughput to a seasonal feedstock, and the requirements of solvent recovery. The most appropriate method depends on the intended application. Supercritical CO_2_ is suited to the recovery of high-value oleoresin where capital investment is available; ultrasound-assisted extraction with food-grade solvents is suited to mid-scale operations for which a low environmental impact is a priority; and pressurized-liquid extraction into a carrier oil is the most direct option where a feed-ready, lycopene-enriched product is required.

**Table 2:** Comparison of conventional and green-extraction technologies for recovery of lycopene and other bioactive compounds from tomato processing by-products. Lycopene yield and key advantages/limitations are summarized from primary technology papers and the comprehensive overview of Eslami et al. [8].

Key Limitations	Key Strengths	Reported Lycopene Yield (Basis as Noted)	Optimal Conditions	Technology	References
Conventional solvent extraction					
Solvent residues; thermal degradation; environmental cost	Simple; high mass recovery	~600–800 mg/100 g oil	Hexane/ethyl acetate, 60–80 °C, 4–8 h	Soxhlet (organic solvent)	[9,65,68]
Supercritical and pressurized-fluid extraction					
High CAPEX; pressure equipment	Solvent-free; food-grade; tunable; biorefinery integration	31.4 mg/100 g DM (90.1% of carotenoids)	≈460 bar, 80 °C	Supercritical CO_2_ (SFE-CO_2_)	[58,59,60,61]
Less polyphenol recovery	Direct lycopene-oil product; feed-ready	44 mg/100 g oil	Sunflower oil as solvent	Pressurized liquid (PLE)	[62]
Energy-assisted physical processes					
Scale-up challenges	Short time; green solvent; low energy	37 mg/100 g peel; 91% yield	600 W ultrasound, ethyl lactate, 30 min	Ultrasound-assisted (UAE)	[62]
Hotspots; sensitive matrix	Rapid heating; high yield	Comparable to UAE	500 W microwave, 0.03 g/mL	Microwave-assisted (MAE)	[62]
Energy-intensive	70% ↑ protein, 32% ↑ polyphenols co-recovery	56.1% lycopene release (water)	10 passes, 100 MPa	High-pressure homogenization (HPH)	[9]
Equipment cost; pretreatment role	Non-thermal; cell-membrane permeabilization	Variable	30 kV/cm, 100 μs pulses	Pulsed electric fields (PEF)	[9]
Green-solvent and biological approaches					
Viscosity; recyclability	Non-toxic solvents; food-grade	Selective carotenoid + tocopherol	Lactic acid:fructose:water; 40 °C	NADES + ultrasound	[63]
Time-consuming; species-specific	Bioconversion to lactic acid; fibre reduction	Enzymatic loosening of cuticle	*L. plantarum*/*S. boulardii*	Solid-state fermentation	[70]

Lycopene yields are reported on heterogeneous denominators that cannot be directly compared across rows: Soxhlet values are per 100 g of recovered oil and the supercritical CO_2_ value is per 100 g of pomace dry matter; UAE and MAE values are per 100 g of fresh peel input; PLE values are per 100 g of carrier oil obtained as the final product; high-pressure homogenization is reported as percent release relative to total peel lycopene content; and NADES and PEF yields are reported on a relative or selective-recovery basis rather than as a unique mass-balance value. CAPEX: capital expenditure; SFE-CO_2_: supercritical CO_2_ extraction; UAE: ultrasound-assisted extraction; MAE: microwave-assisted extraction; PLE: pressurized-liquid extraction; PEF: pulsed electric fields; NADES: natural deep eutectic solvents; ↑: increase.

## 6. Preservation: Drying vs. Ensiling

Fresh tomato pomace contains approximately 75–85% moisture, undergoes rapid microbial spoilage within 24–48 h of generation, and therefore cannot be transported or stored without intervention [9,58]. Two preservation routes dominate. Artificial drying (hot-air, fluidized-bed, or spray drying) produces a stable, dense, easily handled material but adds energy cost and risks thermal degradation of lycopene and tocopherols [71]. Ensiling, that is, controlled lactic-acid fermentation, is energetically cheaper, can be performed on-farm, and preserves a substantial portion of the bioactive load [23,24,69]. The choice between these routes is usually made on the basis of cost and ease of handling, yet their effects on the bioactive fraction differ in ways that have rarely been compared directly. Hot-air drying is the least expensive but the most damaging to lycopene and to the tocopherols, through combined thermal and oxidative degradation; freeze-drying affords the best retention of the carotenoid fraction but is prohibitively costly at feed scale; and spray-drying occupies an intermediate position, with retention strongly dependent on inlet temperature and carrier selection. Ensiling preserves lycopene primarily by excluding oxygen and lowering the pH, although the degree of retention over a complete storage season, and its dependence on the fermentation profile, has received considerably less attention than the microbiological outcome.

The ensiling of tomato pomace was systematically characterized by Wu et al. [23], who preserved pomace for 90 days and isolated 103 lactic acid bacteria (LAB) strains spanning 17 species. The dominant species were *Lactobacillus paracasei* subsp. *paracasei* (35.0% of isolates; now *Lacticaseibacillus paracasei*), *L. harbinensis* (16.5%), *L. plantarum* (10.7%), and *L. manihotivorans* (6.8%) [23]. Over the 90-day storage period the silage pH declined to 4.40, while coliform bacteria were completely inhibited and moulds remained low, consistent with effective control of undesirable microorganisms. Tahmasbi et al. [72] and Abdollahzadeh et al. [73] demonstrated that tomato pomace (dried or ensiled), either alone or mixed with maize silage, retains favourable nutritional value and palatability for ruminants over storage periods exceeding 90 days. Solid-state fermentation with Lactobacillus and Saccharomyces strains further reduces crude fibre, NDF, and ADF while increasing reducing sugars and organic acids [69]. From a circular-economy perspective, ensiling has the major advantage of decentralization: regional processors can deliver wet pomace directly to nearby livestock operations, eliminating drying energy and centralized handling. A further consideration for any preservation route is feed safety. Because the pomace is a moist, sugar-rich matrix, inadequate drying or a poorly controlled fermentation can permit fungal growth and the accumulation of mycotoxins, which have been reported across a range of food-industry by-products and can reach concentrations that warrant dedicated monitoring when such materials are destined for feed [74]. Beyond microbial hazards, by-product streams may also carry pesticide residues and heavy metals originating from the raw crop and its cultivation; routine screening of these contaminants against the relevant feed-safety limits, together with good ensiling practice, is therefore a prerequisite for the safe valorization of tomato pomace in animal diets [75].

## 7. Application in Animal Nutrition

The animal-feeding literature on tomato pomace and lycopene is now sufficiently developed to permit some cross-species generalization, although each species responds to the matrix differently. Poultry, for which the largest and most coherent body of evidence is available, is considered first. The foundational work on heat-stressed Japanese quails and broilers by Sahin and co-workers [21,52,53,76] established what has since become a consistent pattern: dietary lycopene in the 100–400 mg/kg range or tomato powder at 25–50 g/kg suppresses hepatic NF-κB, upregulates Nrf2 and its antioxidant response targets, lowers malondialdehyde, raises serum SOD and GPx activities, and partially restores growth performance under cyclic 34 °C thermal challenge. Hosseini-Vashan et al. [77] confirmed the same observation using dried pomace at 5% rather than purified lycopene, with the additional finding of improved blood lipid profile and reduced abdominal fat deposition. More recent broiler studies have added molecular detail (Wang et al. [16] showing direct upregulation of hepatic Nrf2, SOD2, NQO1, and HO-1 mRNA at 30 mg/kg lycopene), the meat-quality dimension (the 2024 report of Wu et al. describing reduced drip loss, improved colour, and *Lactobacillus*-favouring shifts in cecal microbiota [78]), and the contaminant-mitigation angle (Ma et al. [79] showing protection against aflatoxin B1-induced muscle damage at 200 mg/kg). Yang et al. [80] integrated cecal metagenomics with hepatic untargeted metabolomics in yellow-feathered broilers and tied the growth-performance recovery during heat stress to coordinated reorganization of cecal microbial composition and hepatic lipid and amino-acid metabolism. Across this body of work, the most robust signal is the antioxidant–inflammatory dual modulation under thermal challenge, with the dose-dependent behaviour suggesting an effective range of roughly 30 to 400 mg/kg purified lycopene or 5–10% pomace. This body of poultry evidence should nonetheless be interpreted in the light of several methodological constraints. Most trials are of short duration and use modest group sizes, and the form of supplementation varies between purified lycopene and whole or powdered pomace; a dose expressed as milligrams of lycopene per kilogram of feed is therefore not strictly comparable from one study to another. The representative in vivo studies discussed in this section are summarized in Table 3.

The evidence in laying hens and quails follows a somewhat different pattern. In these species, the mechanism of primary interest is not antioxidant rescue under stress, but rather the transfer of dietary lycopene through the hen into the egg. At dietary inclusion levels of 10–20 mg/kg lycopene or approximately 17 g/kg tomato paste, yolk concentrations of lycopene and β-carotene increase measurably, serum and yolk cholesterol decrease, and the oxidative stability of stored eggs is improved [55,56,81,82]. The transfer rate to yolk lipid has been reported at approximately 0.13–4.5% of dietary lycopene [56,81]; although modest, this level is sufficient to establish a functional-food link from an agro-industrial by-product, through the hen, to the human consumer. The 12% pomace inclusion study of Reda et al. [83] in quail breeders documented improved productive and reproductive performance, blood metabolites, and carotenoid deposition into egg yolk, reinforcing this functional-food link.

Ruminants are the other species class where the evidence is genuinely well-developed, and the role of pomace there is conceptually different again. Here the matrix is not an additive but a substantial dietary component, partly replacing forage and concentrate while carrying lycopene and other bioactives along with the fibre. Tuoxunjiang et al. [84] replaced 10% of maize silage with ensiled tomato pomace in lactating Holstein cows and observed higher dry matter intake and digestibility without depression of milk yield, alongside improvements in milk vitamin concentrations and peripartum antioxidant and immune indices [85]. Ebeid et al. [86] reported the same outcome pattern in lactating Egyptian buffaloes, with additional documentation of higher fat-corrected milk yield, milk fat percentage, and milk PUFA content following pomace silage feeding. Correddu et al.’s systematic synthesis of grape, pomegranate, olive, and tomato by-products in dairy ruminants [87] places tomato pomace squarely within an emerging consensus: by-product bioactives carry over to milk, enhance the dairy product nutraceutical profile, and do not consistently depress milk production. In growing lambs and goats, Fondevila et al. [88] showed that diets containing up to 300 g/kg pomace did not reduce intake or daily gain, consistent with the practical 40% cap derived from broader synthesis [9], with 200 g/kg matching soybean-protein performance. In dairy goats, the milk-yield effect is similarly neutral while milk quality and fat content improve [89]. Beef-cattle work has shown that rumen fermentation indices shift modestly under 3–11% pomace inclusion without measurable effect on intake or nitrogen retention [90]. The hypothesis that the fibre-and-polyphenol combination supports methane reduction, by analogy with the olive, grape, and pomegranate literature, is plausible and untested at sufficient scale; direct in vivo methane data with proper dose–response coverage are not yet available. Pouraghakouchak et al. [91] add a calf-specific nuance: 100 g/kg tomato pomace alone did not improve—and overall slightly impaired—starter-phase growth performance, while the reduction in blood malondialdehyde was driven by the flaxseed oil rather than by the pomace; a significant pomace × flaxseed-oil interaction meant that the combination, with its lower n-6:n-3 ratio, outperformed either ingredient alone, indicating that the value of pomace in calves lies in pairing its antioxidant cargo with a favourable fatty-acid background rather than in any standalone growth benefit.

The evidence available for pigs is the most equivocal. Correia et al. [60] reported no improvement in meat colour, fatty acid profile, or detectable carotenoid deposition in muscle following 5% pomace inclusion in young pigs, although increased α-tocopherol concentrations in meat and liver and an improvement in the oxidative stability of stored meat were observed. A converging conclusion was reached by An et al. [57] in finishing pigs, in which dietary lycopene at 20 mg/kg reduced MDA concentrations in pork belly without altering the plasma lipid profile. Taken together, these findings suggest that the dominant effect of lycopene in swine is tissue-level oxidative protection rather than systemic carotenoid deposition. The relatively poor intestinal absorption of intact dietary carotenoids in monogastric species, together with the digestibility limits imposed by the high-fibre matrix at inclusion levels above 5%, constrain broader application of tomato pomace in this species. By contrast, rabbits have recently emerged as a focus of considerable interest. The 2024 trial of Hassan et al. [92] in New Zealand White rabbits, conducted under Mediterranean summer conditions, evaluated 0.5, 1.0 and 1.5% dried pomace powder and reported the highest growth rate at 1.5% inclusion, alongside the lowest feed intake, together with improvements in meat quality and lipid-health indices. Earlier studies at higher inclusion levels have documented enhanced immune responses, including increased immunoglobulin levels, phagocytosis and chemotaxis [93]. The geographical co-distribution of tomato processors and meat-rabbit production across Mediterranean smallholder systems, together with the species’ particular sensitivity to summer heat stress, identifies the rabbit as a particularly suitable target for pomace integration in regional circular-feed schemes. When the species are compared directly, a consistent pattern emerges that is less apparent from the study-by-study description. Ruminants tolerate and benefit from pomace as a bulk dietary component, because rumen fermentation accommodates the high-fibre matrix and because carotenoids partition efficiently into milk fat. Poultry respond principally through the antioxidant and anti-inflammatory pathways under heat or mycotoxin challenge, and deposit measurable lycopene in egg yolk and muscle. Pigs show oxidative protection at the tissue level but little carotenoid deposition, a response limited by the poor intestinal absorption of intact carotenoids and by the constraints that the high-fibre matrix imposes on digestibility above approximately 5% inclusion. Rabbits, as hindgut fermenters, appear well suited to the matrix at low inclusion levels. These differences therefore reflect digestive physiology, that is, whether the animal is a ruminant, a hindgut fermenter, or a simple monogastric, together with the associated differences in fibre tolerance and carotenoid bioavailability, rather than any species-specific action of lycopene.

**Table 3:** Summary of representative in vivo studies on tomato pomace and lycopene supplementation across livestock species.

Direction	Key Outcomes	Inclusion Level	Supplementation Mode	Species/Class	References
Beneficial	↑ Antioxidant enzymes (SOD, GPx); ↓ MDA; heat-stress mitigation; meat oxidative stability	5–10%	Pomace (dried)	Broiler chicken	[76,77,79]
Beneficial	Nrf2 induction; ↓ MDA; thermal-stress recovery; aflatoxin protection	30–400 mg/kg	Purified lycopene	Broiler chicken	[18,23,52,53,78]
Beneficial	↑ Yolk lycopene, β-carotene, lutein; ↓ yolk MDA after storage	5–17 g/kg	Tomato powder	Layer hen	[55,80]
Beneficial	↑ Yolk lycopene & colour; ↓ serum & yolk MDA; ovarian Nrf2/HO-1 activation	10–20 mg/kg	Purified lycopene	Layer hen	[50,56]
Beneficial	↑ Egg production, immune response; ↓ liver MDA	3–12%	Pomace (dried)	Japanese quail	[82]
Beneficial	↑ Antioxidant status; performance maintained under heat stress	25–100 mg/kg	Tomato powder/purified lycopene	Japanese quail	[52,73,81]
Beneficial/neutral on yield	↑ DMI, digestibility, milk vit; antioxidant/immune indices; no milk-yield depression	10% (replacing maize silage)	Ensiled pomace	Dairy cow	[83,86,94]
Beneficial	↑ 7–FCM, fat %, PUFA content; no yield depression	TMR fraction	Pomace silage (TMR)	Lactating buffalo	[84]
Neutral on growth	No effect on ADG/DMI	Up to 300 g/kg DM; commercial 5–30%	Pomace (dried or fresh)	Sheep/Lamb	[87]
Beneficial on milk quality	No effect on yield; improved milk quality, fat content	Up to 40% DM	Pomace	Goat (dairy)	[88]
Neutral	No effect on intake; altered rumen fermentation; no detriment to N retention	3.2–11.2%	Dried pomace (TMR)	Beef steer	[89]
Mixed (synergy with flaxseed oil)	No growth benefit from pomace alone; benefit only via pomace × flaxseed-oil synergy; ↓ LDL	Starter inclusion	Pomace + n-6:n-3 control	Dairy calf (starter)	[90]
Beneficial on oxidative stability	↑ α-tocopherol in meat/liver; ↓ MDA in belly meat; no fatty acid or cholesterol changes	3–5%/20 mg/kg	Pomace 3–5%/lycopene 20 mg/kg	Finishing pig	[57,91]
Beneficial	↑ Growth (esp. 1.5%); improved meat quality; improved lipid-health indices	0.5–1.5%	Dried pomace powder	Rabbit (NZW)	[92,93]

↑: increase; ↓: decrease.

## 8. Circular Economy Integration

The valorization of tomato by-products in animal nutrition aligns with multiple converging policy frameworks: the European Green Deal, the EU Farm-to-Fork Strategy, the Sustainable Development Goal 12.3 on halving per-capita global food waste by 2030, and the proposed EU Circular Economy Act [3,27,95,96]. From a systems perspective, the tomato pomace value chain exemplifies the cascade-use principle: highest-value molecular fractions (lycopene oleoresin, tomato seed oil for cosmetics and nutraceuticals) are recovered first by green extraction; the residual de-oiled, de-pigmented matrix retains substantial protein, fibre, and minerals and is then channelled to livestock feeding either directly (drying) or after ensiling [6,8,65]. Animal manure subsequently re-enters the agronomic loop as organic fertilizer for tomato cultivation, closing the nutrient cycle. An important qualification applies at this point, since much of the circular-economy argument for tomato pomace still rests on evidence extrapolated from other agro-food systems rather than on tomato-specific analyses. Several of the life-cycle and profitability figures presented below are derived from studies of grape, olive, or mixed residues, or from sector-wide modelling. The number of assessments that isolate tomato pomace within a defined animal-production system remains small, and this distinction should be borne in mind when the environmental and economic benefits are quantified.

Three quantitative arguments support the circular-feed case for tomato pomace. First, life-cycle assessments by Eslami et al. [6] and biophysical modelling by van Selm et al. [29] indicate that the marginal greenhouse gas intensity of feed produced from co-products is substantially lower than that of dedicated feed crops, owing to attributional allocation across multiple product streams. Second, the European feed industry has demonstrated that practically all compound-feed raw materials are already non-food-grade [4], establishing institutional feasibility; tomato pomace integration extends this baseline rather than challenging it. Third, in regional production systems such as those of Türkiye (20% processing rate), Italy (12.2% of global processed output), Spain, Greece, and Tunisia, tomato processors and livestock enterprises are geographically concentrated within Mediterranean agro-ecosystems. This concentration creates short logistics chains that are particularly suited to the perishable wet pomace stream, which can be ensiled at the processor or transported within 48 h to nearby livestock operations [6,24].

Beyond environmental indicators, the circular framing introduces socio-economic dimensions. The Ellen MacArthur Foundation has projected that EU-wide adoption of circular food systems could increase farm profits by up to USD 3100 per hectare and lower agricultural greenhouse gas emissions by approximately 70% [97]. For tomato processors, by-product valorization turns pomace from a disposal cost (landfill, transport, and tipping fees) into a revenue-generating co-product, with reported positive net present values and short payback times for biorefinery investments at industrial scale [62]. The proposed Circular Economy Act would further institutionalize this transition by creating a single market for secondary inputs and reducing regulatory bottlenecks that currently impede higher levels of feed circularity in the EU [96]. A preliminary assessment of economic feasibility can be based on the balance among three terms: the disposal cost that is avoided, which comprises landfill and tipping fees; the market value of the recovered high-value fractions, principally lycopene oleoresin and seed oil; and the capital and energy cost of the extraction and preservation chain. Published net-present-value analyses of supercritical-CO_2_ recovery indicate that this balance is favourable at industrial throughput [62,97], although plant-scale estimates specific to feed-grade tomato pomace remain scarce and constitute a clear objective for future techno-economic analysis.

## 9. Limitations and Future Directions

The literature reviewed here is substantial but its limitations should be stated explicitly. The most consequential is the heterogeneity of tomato pomace itself. Cultivar (for example San Marzano, Roma, and processing-specific hybrids), processing technology (cold-break versus hot-break, and the presence or absence of finishers and deseeders), and downstream handling (whether the residue is delivered wet, ensiled, dried, fermented, or defatted) each contribute to the final composition of the residue. The resulting variation in lycopene, polyphenol, and fibre content is rarely standardized in published feeding trials [9,31]. Without that standardization, meta-analytic synthesis is structurally difficult, and the field would benefit substantially from routine reporting of HPLC-quantified lycopene including explicit E/Z-isomer ratios, total phenolic content, and in vitro digestibility. These requirements may be distinguished usefully according to the type of work that each entails. The adoption of a common characterization standard, comprising baseline HPLC determination of lycopene with its E/Z-isomer ratios, total phenolic content, proximate composition, and a description of processing conditions, represents an immediate reform of reporting practice. Dose–response and bioavailability studies, together with investigation of the interaction between pomace bioactives and the gut microbiota, require controlled experimental work. Validation under commercial production conditions, and life-cycle and cost–benefit assessment specific to tomato pomace, belong to the subsequent stage of scale-up. Addressing these needs in this sequence would allow the evidence base to develop in a systematic rather than a fragmentary manner.

The species-dependence of lycopene deposition is a second open question. Ruminants and poultry transfer measurable lycopene to milk, eggs, and muscle; monogastric pigs show inconsistent deposition despite documented tissue antioxidant effects [57,60]. A different mechanistic basis—differential expression of intestinal carotenoid transporters such as SR-BI, CD36, and NPC1L1, and possibly differential bile micelle dynamics—is suspected but not characterized. Closely related to this is the absence of well-mapped dose–response curves in any species. The reported effective ranges, from 5% to 40% pomace and from 20 mg/kg to 400 mg/kg purified lycopene, cover nearly an order of magnitude. This is less a problem of biology than of unstandardized practice in the absence of regulatory frameworks or commercial inclusion limits. Other gaps follow from the same pattern. The interaction between tomato pomace bioactives and rumen microbial ecology has been hypothesized from the olive, grape, and pomegranate by-product literature [87,98] but not directly tested in vivo at meaningful scale; the possibility of methane reduction via polyphenol-tannin pathways is of considerable interest and is one of the more obvious openings for the next round of ruminant work. Life-cycle assessments specific to tomato pomace as feed are scarce; the broader by-product valorization LCA literature [6] needs species-specific extensions that account for the full animal-source food chain. Translation to commercial feed-grade lycopene oleoresin at scale will require not only the green-extraction technology, which is now available, but also economic modelling that compares the cascade biorefinery to conventional feed inputs in the production economics of a regional Mediterranean operation. Regulatory harmonization through EFSA and Codex Alimentarius would lower the activation energy for cross-border trade in co-product-derived feeds. Finally, the climate-change dimension deserves direct attention: thermal challenge experiments in poultry, dairy, and rabbits under realistic Mediterranean summer conditions would provide the empirical foundation for the heat-stress-mitigation argument that the present review places at the conceptual centre of the case for pomace valorization.

## 10. Conclusions

Tomato processing by-products represent one of the most chemically valuable and quantitatively important agro-industrial residue streams in the global food system. The combination of lycopene (36.7–50.2 mg/100 g DM), polyphenols (~161.8 mg GAE/g), tocopherols, dietary fibre, seed oil, and protein constitutes a multi-fraction matrix that is particularly well suited to cascade-biorefinery valorization. The molecular mechanisms underlying the antioxidant and anti-inflammatory effects of lycopene, namely singlet-oxygen quenching, Keap1–Nrf2–ARE activation, NF-κB suppression, and modulation of lipid metabolism, translate into measurable production responses in poultry, ruminants, pigs, and rabbits, with the strongest evidence to date observed in heat-stressed poultry and in ruminant milk quality. Ensiling represents an economically and ecologically attractive preservation route that maintains bioactive integrity while enabling regional integration of tomato processors with livestock operations. Taken together, several factors position tomato by-product valorization as an exemplary case study of circular-feed implementation: the European Green Deal, the Circular Economy Act, the climate-change-driven heat-stress burden on livestock, and the geographical concentration of both tomato processing and animal production within the Mediterranean basin. Standardized quantification of bioactive components, more clearly defined inclusion thresholds, life-cycle assessment, and regulatory harmonization are likely to remain the principal research and policy priorities over the coming decade.

## Figures and Tables

**Table 1:** Macronutrient, mineral, and bioactive composition of tomato pomace and its main fractions (per kg dry matter unless otherwise stated) *.

Pulp/Other	Seed	Peel	Whole Pomace	Component	References
Proximate composition (g/kg DM)					
~120	230–250	~150	150–230	Crude protein	[10,12,30]
~15	~200	~25	50–200	Crude fat	[10,13,30]
~50	~40	~40	40–60	Ash	[8,10,30]
~310	~410	~570 (SDF ≈ 89; IDF ≈ 485)	250–510	Total dietary fibre	[10,14,16]
Minerals (g/kg DM)					
NR	NR	NR	~13.1	Potassium (K)	[30]
NR	NR	NR	~2.8	Phosphorus (P)	[30]
Carotenoids (mg/100 g DM)					
~38	~5	~170	36.7–50.2	Lycopene	[10,14,30]
~1.5	trace	~1.5	NR	β-carotene	[10,30]
Phenolic compounds and antioxidant capacity					
~33	~18	~37	94.5–213.4	Total phenolics (mg GAE/g DM)	[10,30]
moderate	low	high	30.6–378.7	Total flavonoids (mg QE/g DM)	[10,30]
NR	NR	NR	29.9–75.0	DPPH radical scavenging (%)	[9]
Fatty acid profile					
NR	high	NR	55.7–62.3	PUFA (% of total fatty acids)	[30]

* Mean values and ranges compiled from major reviews [10,30] and primary chemical analyses [12,13,14,15,16,30]. Values are compiled from heterogeneous source studies and are not strictly fraction-weighted. Whole-pomace lycopene (36.7–50.2 mg/100 g) reflects published surveys of dried whole pomace [9], whereas peel (~170 mg/100 g) and pulp (~38 mg/100 g) reflect fraction-specific analyses [12]; differences in cultivar, processing route, and analytical denominator (extract vs. dry matter basis) explain why the whole-pomace range does not sit between the fraction values as a simple weighted mean. The same caveat applies to total phenolics: the whole-pomace range (94.5–213.4 mg GAE/g) is drawn from phenolic-rich extracts and concentrate fractions reported in [10,30], while peel, seed, and pulp values are reported on a dry-matter basis from primary chemical analyses; the columns are therefore comparable within their analytical category but not across them. NR: not reported. SDF: soluble dietary fibre; IDF: insoluble dietary fibre; GAE: gallic acid equivalents; QE: quercetin equivalents.

## Data Availability

No new data were created or analyzed in this study. Data sharing is not applicable to this article.

## Abbreviations

The following abbreviations are used in this manuscript:

ACC	Acetyl-CoA carboxylase
ADF	Acid detergent fibre
APC	Article Processing Charge
ARE	Antioxidant response element
CAPEX	Capital expenditure
CPT1	Carnitine palmitoyltransferase 1
DM	Dry matter
DMI	Dry matter intake
DPPH	2,2-Diphenyl-1-picrylhydrazyl
EU	European Union
FAS	Fatty acid synthase
FCM	Fat-corrected milk
FEFAC	European Feed Manufacturers’ Federation
GAE	Gallic acid equivalents
GCL	γ-Glutamylcysteine ligase
GPx	Glutathione peroxidase
HDL-C	High-density lipoprotein cholesterol
HO-1	Heme oxygenase 1
HPLC	High-performance liquid chromatography
Keap1	Kelch-like ECH-associated protein 1
LAB	Lactic acid bacteria
LCA	Life-cycle assessment
MAE	Microwave-assisted extraction
MDA	Malondialdehyde
NADES	Natural deep eutectic solvents
NDF	Neutral detergent fibre
NF-κB	Nuclear factor kappa B
NQO1	NAD(P)H quinone dehydrogenase 1
Nrf2	Nuclear factor erythroid 2-related factor 2
PEF	Pulsed electric fields
PLE	Pressurized liquid extraction
PPARα	Peroxisome proliferator-activated receptor alpha
PUFA	Polyunsaturated fatty acids
QE	Quercetin equivalents
ROS	Reactive oxygen species
SDF	Soluble dietary fibre
SDG	Sustainable Development Goal
SFE-CO_2_	Supercritical carbon dioxide extraction
SOD	Superoxide dismutase
TMR	Total mixed ration
UAE	Ultrasound-assisted extraction
